# Minimally invasive elective gastrectomy after preoperative chemotherapy in a patient with frailty who presented with locally far advanced-stage gastric cancer: a case report

**DOI:** 10.1186/s40792-024-01942-6

**Published:** 2024-06-13

**Authors:** Naoto Shirakami, Shingo Kanaji, Atsushi Shimada, Tomosuke Mukoyama, Ryuichiro Sawada, Hitoshi Harada, Tomonori Tanaka, Naoki Urakawa, Hironobu Goto, Hiroshi Hasegawa, Kimihiro Yamashita, Takeru Matsuda, Yoshihiro Kakeji

**Affiliations:** 1 Division of Gastrointestinal Surgery, Department of Surgery, Graduate School of Medicine, Kobe University, 7-5-2, Kusunoki-Cho, Chuo-Ku, Kobe, Hyogo 650-0017 Japan; 2 Department of Diagnostic Pathology, Graduate School of Medicine, Kobe University, Hyogo, Japan

**Keywords:** Gastric cancer, Multiple invasions of other organs, Poor general condition, Older patients, Preoperative chemotherapy, Laparoscopic distal gastrectomy

## Abstract

**Background:**

Herein, we report a case of gastric antrum cancer with multiple invasions to other organs that was completely cured with laparoscopic distal gastrectomy after preoperative chemotherapy in a patient with poor general condition.

**Case presentation:**

An 80-year-old male patient was diagnosed with anemia during follow-up for cerebral lacunar infarction at another hospital. He was diagnosed with advanced-stage gastric antrum cancer and was referred to our hospital. On esophagogastroduodenoscopy, type 2 advanced-stage gastric cancer was detected at the greater curvature of the antrum, and the biopsy results revealed tubular adenocarcinoma. Contrast-enhanced computed tomography scan revealed multiple invasions to other organs, thick gastric wall with contrast effect, and superior mesenteric vein tumor thrombus. However, there was no evidence of distant metastasis on positron emission tomography/computed tomography scan. The clinical diagnosis was stage IVA gastric cancer. Pancreatoduodenectomy with portal vein resection could be important at this point. However, preoperative chemotherapy with S-1 and oxaliplatin was administered instead of performing extended surgery because the patient had poor general condition (performance status score of 3). The patient received three cycles of preoperative chemotherapy at the hospital along with rehabilitation and nutritional management with oral nutritional supplements. After treatment, the performance status score of the patient improved from 3 to 1. Furthermore, in terms of clinical therapeutic effect, the patient achieved partial response. Hence, laparoscopic distal gastrectomy with D2 lymph node dissection and partial transverse colectomy was performed. After surgery, the patient was admitted for oral intake on postoperative day 6 and was discharged on postoperative day 21. Based on the histopathological examination, gastric cancer had disappeared, and there were no evident malignant findings. Therefore, gastric cancer was classified as grade 3 according to the histological treatment efficacy criteria. The patient did not present with recurrence at 2 years after surgery.

**Conclusions:**

By actively administering preoperative chemotherapy, minimally invasive radical surgery with maximum preservation of the surrounding organs can be performed for locally far advanced-stage gastric cancer in older patients with poor general condition.

## Background

Gastric cancer is one of the most common malignancies with a high incidence rate in Japan. The number of patients is second most followed by prostate cancer in males and fourth most in females according to the statistics in 2017 [1]. The number of gastric cancer cases decreased worldwide due to the lower prevalence of *Helicobacter pylori* infection, which is attributed to its eradication. However, in Japan, the incidence rate of gastric cancer remains high, and the peak age is in the 80 s [2, 3]. To date, in Japan, approximately half of gastric cancer cases were detected at an early stage. Meanwhile, various minimally invasive treatments such as endoscopic resection are currently developed, and highly invasive surgical procedures are often important for advanced-stage cancers. Recently, some studies have assessed the use of multidisciplinary therapy such as perioperative chemotherapy for advanced-stage gastric cancer with multiple invasions to other organs. However, gastric cancer still has a poor prognosis [4]. Elderly patients with a poor general condition due to far advanced-stage gastric cancer with multiple invasions to other organs do not commonly undergo chemotherapy and surgical operation. In some cases, the best supportive care is considered. However, preoperative chemotherapy is administered within a short time before surgery. Moreover, it can be more useful in elderly patients than postoperative chemotherapy, which is often unsuccessful due to multiple postoperative complications and poor organ function and performance status. The percentage of elderly patients aged > 75 years in Japan can increase to > 60% within a decade. Thus, gastric cancer treatment in older patients, particularly those with poor general condition, should be actively discussed. Herein, we report a case of gastric antrum cancer with multiple invasions to other organs that was completely treated with laparoscopic distal gastrectomy after preoperative chemotherapy in an older patient with poor general condition.

## Case presentation

An 80-year-old male patient presented with anemia during follow-up for cerebral lacunar infarction at another hospital. He was diagnosed with gastric antrum cancer on esophagogastroduodenoscopy (EGD). Then, he was referred to the gastroenterology department of our hospital due to his contrast-enhanced computed tomography (CECT) scan findings. In particular, he had aortic valve stenosis and paroxysmal atrial fibrillation, similar to other medical histories, and he took anticoagulant drugs. His American Society of Anesthesiology Physical Status score worsened from 0 to 3 within a few months as the cancer progressed. In addition, as his general condition declined, his food intake also became poor.

**Fig. 1:**
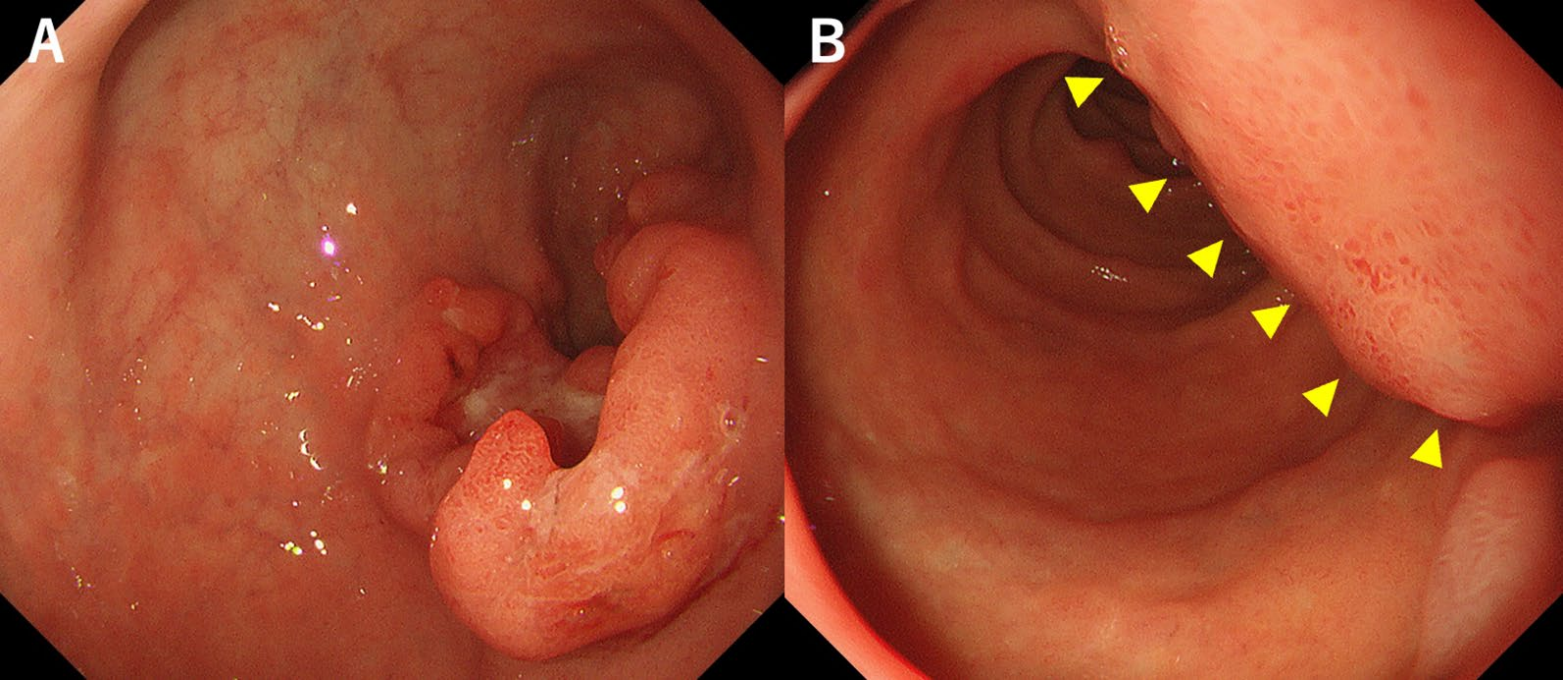
Esophagogastroduodenoscopy showed type 2 advanced-stage gastric cancer at the greater curvature of the antrum (**A**) and the extramural invasion of the descending part of the duodenum (**B**)

**Fig. 2:**
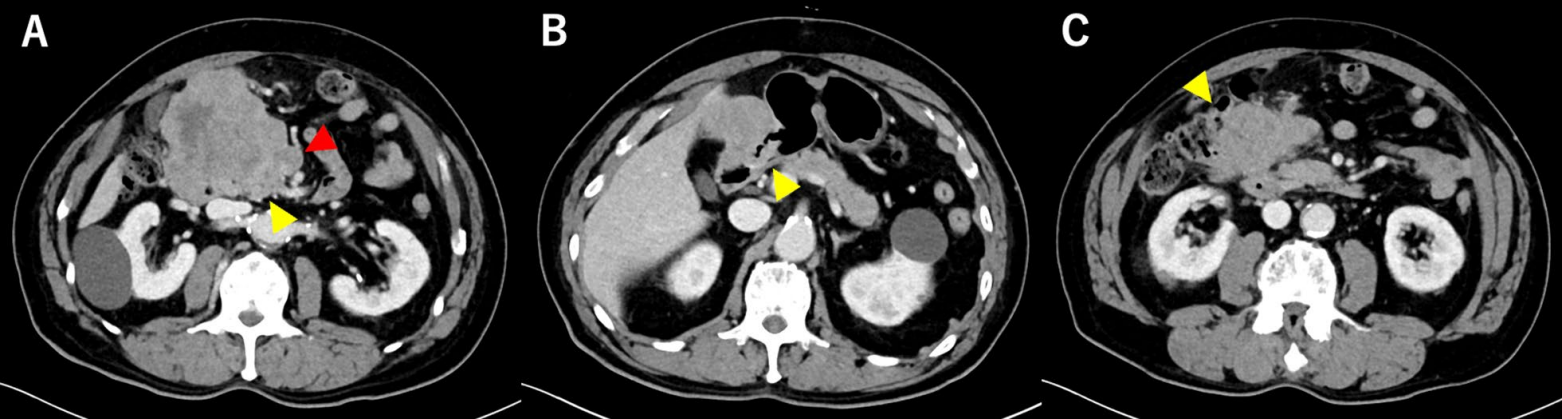
Contrast-enhanced computed tomography scan revealed a huge mass clumped with the swollen infrapyloric lymph node, invasion to the pancreatic head (**A**, yellow triangle), thick gastric wall with contrast effect (**B**), invasion to the descending part of the duodenum and transverse colon (**C**), and a continuous shadow defect from the tumor located in the superior mesenteric vein (**A**, red triangle)

**Fig. 3:**
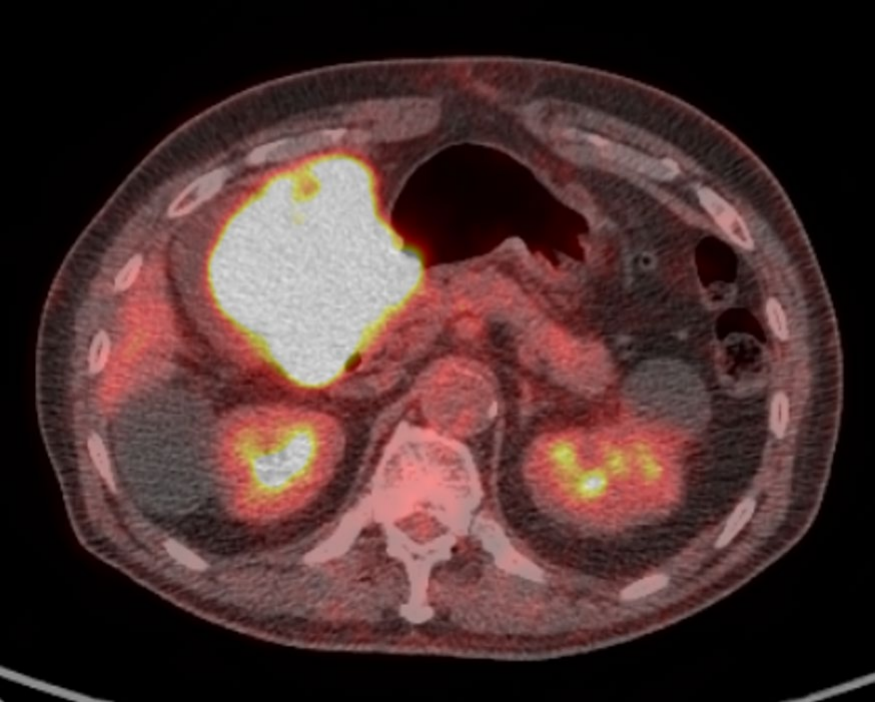
Positron emission tomography/computed tomography scan showed an evidently elevated ^18^F-fluorodeoxyglucose uptake in the lesion site including the tumor thrombus

Laboratory data showed slight anemia (hemoglobin level at 11.7 g/dL) and moderate elevation of inflammatory markers (white blood cell count at 9500/µL and C-reactive protein level at 0.36 mg/dL). There were no evident abnormalities in liver or renal function. The patient’s serum carcinoembryonic antigen level was high at 8.6 ng/mL. Meanwhile, his carbohydrate antigen 19–9 level was normal (6 U/mL). EGD showed type 2 advanced-stage gastric cancer at the greater curvature of the antrum (Fig. 1A) and extramural invasion of the descending part of the duodenum (Fig. 1B). Biopsy was performed, and results showed tubular adenocarcinoma, and HER2 status was negative. The CECT of the thorax and abdomen presented with a huge mass clumped with swollen infrapyloric lymph node with a size of approximately 9 cm (Fig. 2A) and thick gastric wall with contrast effect (Fig. 2B). The tumor invaded the pancreatic head (Fig. 2A), the descending part of the duodenum, and the transverse colon (Fig. 2C). Moreover, it had a continuous shadow defect from the tumor located in the superior mesenteric vein (Fig. 2A). Based on this finding, the patient was diagnosed with superior mesenteric vein tumor thrombus. Positron emission tomography/computed tomography (PET/CT) scan revealed evidently elevated ^18^F-fluorodeoxyglucose (FDG) uptake in the lesion site, including the tumor thrombus. However, there was no evidence of distant metastasis other than that in the infrapyloric lymph node (Fig. 3). The clinical diagnosis was gastric cancer L Gre-Post tub cType2 cT4b (pancreas, duodenum, and transverse colon) N + M0, cStageIVA according to the Union for International Cancer Control Tumor, Node Metastasis Classification of Malignant Tumors, Eighth Edition. The lymph node station was defined using the Japanese Classification of Gastric Cancer, Fifteenth Edition. Although pancreatoduodenectomy with portal vein resection is important at this point, pancreatoduodenectomy could not be performed as the patient’s general condition was poor (performance status [PS] score of 3). If the patient’s general condition was attributed to tumor progression, chemotherapy could be considered, and he could be treated with chemotherapy with S-1 and oxaliplatin (SOX) based on curability and his general condition. The patient was HER2 negative, and SOX was selected as the regimen in the hope that oral intake would improve his general condition by using the intestinal tract.

**Fig. 4:**
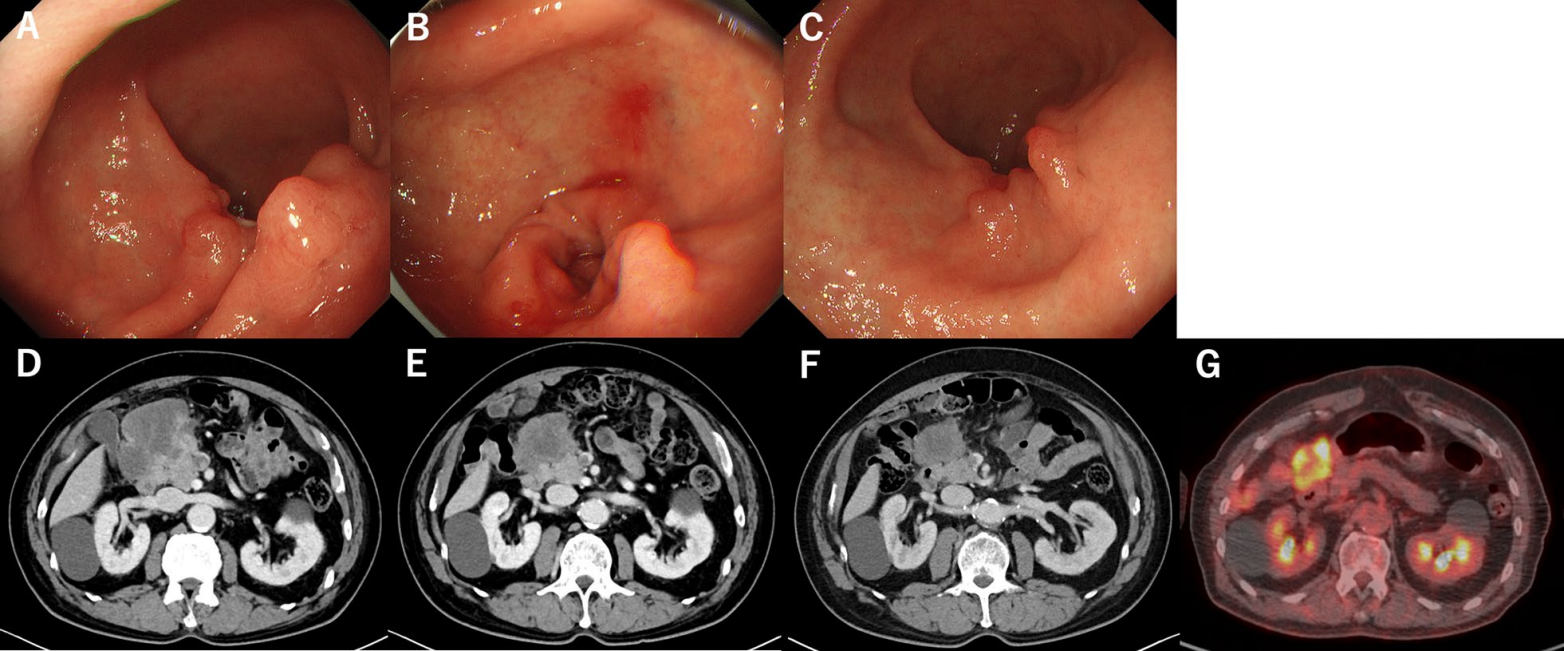
After three courses of SOX, the tumor had shrunk on esophagogastroduodenoscopy (**A**–**C**) and contrast-enhanced computed tomography scan (**D**–**F**). Positron emission tomography/computed tomography scan revealed that the FDG uptake in the tumor was decreasing (**G**)

S-1 was administered orally twice daily for 2 weeks, followed by a 1-week rest. The S-1 dose was determined based on the body surface area as follows: < 1.25 m^2^, 80 mg/day; 1.25 to < 1.50 m^2^, 100 mg/day; and > 1.50 m^2^, 120 mg/day. In this case, S-1 was initiated with a dose of 100 mg/day based on the patient’s BSA that was between 1.25 and 1.50 m^2^. However, the patient had loss of appetite on day 4. Thus, the S-1 dose was reduced by one step to 80 mg/day. Oxaliplatin was administered intravenously at 130 mg/m^2^ on day 1. During this regimen, no severe adverse events, except for loss of appetite, according to the National Cancer Institute Common Terminology Criteria for Adverse Events version 5.0. were detected. All three cycles of preoperative chemotherapy along with rehabilitation and nutritional management with oral nutritional supplements were performed at the hospital. It improved the patient’s performance status score from 3 to 1. After three cycles of SOX, the patient’s CEA showed improvement over time. The tumor observed at the greater curvature of the antrum on EGD had shrunk (Fig. 4A–C). In CECT, the tumor shrunk by approximately 90% after one cycle and by approximately 50% after three cycles (Fig. 4D–F). The clinical therapeutic effect was classified as partial response on radiological examination according to the Response Evaluation Criteria in Solid Tumors criteria version 1.1. Moreover, the border between the tumor and pancreas was partially obscured, however, much clearer than before chemotherapy. PET/CT scan showed that the FDG uptake in the tumor decreased, and the FDG uptake in the tumor thrombus was not prominent (Fig. 4G). After chemotherapy, the condition was diagnosed as ycT4b (transverse colon) N + M0 and cStage IVA. As the patient’s ASA-PS score improved and the FDG uptake on the tumor thrombus was not prominent, we planned to perform laparoscopic distal gastrectomy with D2 lymph node dissection and partial transverse colectomy.

## Operative technique

**Fig. 5:**
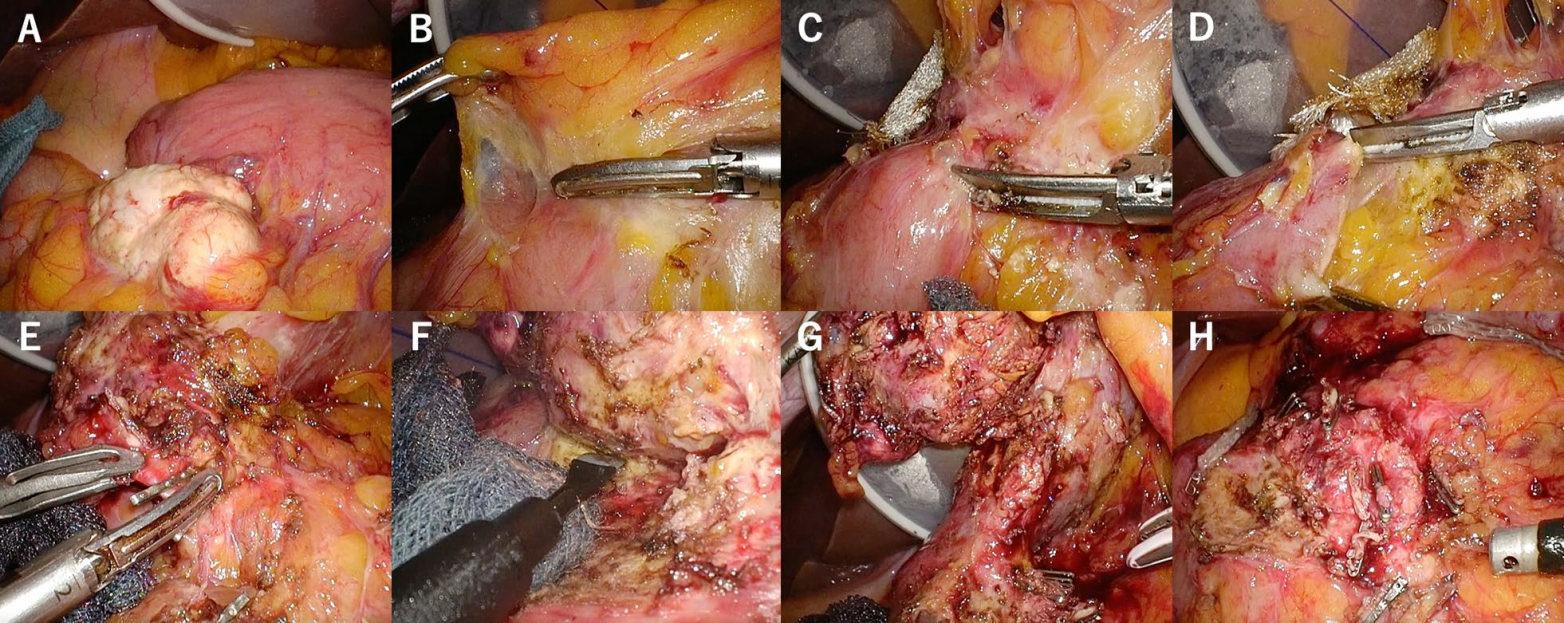
Infrapyloric lymph node became white tone and swollen (**A**) and strongly adhered to the transverse colon and the anterior surface of the pancreas due to the effect of chemotherapy or invasion. We performed the ablative procedure between the tumor and the transverse colon (**B**–**D**). By the procedure, we could identify the anterior surface of the pancreas. The tumor was dissected from the pancreas in the same way as the dissection of the transverse colon (**E**, **F**). After we performed the ablative procedure, the duodenum was resected (**G**, **H**)

We started the surgery in the open leg position under general anesthesia. A 12-mm camera port was inserted at the umbilical incision, and AirSeal^®^ (ConMed, Largo, FL, USA) was used for insufflation. A 5-mm port was placed in the right upper abdomen, and three 12-mm ports were placed in the right lateral, left upper, and left lateral abdomen. There were no evident findings indicating liver metastasis or peritoneal dissemination in the abdominal cavity. The intraoperative rapid ascites cytology result was negative. The infrapyloric lymph node became white and swollen (Fig. 5A) and invaded the greater omentum and transverse colon (Fig. 5B). To perform the complicated resection of the transverse colon with invasion, the blood vessels nourishing the transverse colon were dissected (Fig. 5C, D). Furthermore, the right branch of the median colon artery was also entrapped in the tumor. Hence, the artery at the root was resected. Via this procedure, the border between the tumor and the pancreas could be recognized, and the vessel that was believed to be the right gastroepiploic artery and vein was identified (Fig. 5E). The tumor strongly adhered to the anterior surface of the pancreas due to the effect of chemotherapy or invasion. Therefore, ablation was performed by attaching the pancreatic capsule to the tumor side or partially cutting into the pancreas (Fig. 5F). We had considered the possibility of tumor invasion into the pancreas, however, did not plan to go as far as pancreaticoduodenectomy, because we thought that it would be too invasive considering his PS. After vascular resection and infrapyloric lymph node dissection, the duodenum in the 4-cm distal area was resected from the pyloric ring because of tumor invasion into the duodenum using Signia (color: purple, size: 60 mm; Covidien, Mansfield, MA, the USA) (Fig. 5G, H). After resecting the left side of the great omentum and lymph node dissection, suprapancreatic lymph node dissection was performed. After the lesser curvature lymph node dissection, the gastric wall was cut from the greater curvature side using Signia twice, and the gastric specimen was collected. Intraoperative rapid histopathological examination showed that the oral and anorectal resection margins were negative. Subsequently, mobilization of the right hemicolon from the retroperitoneum was performed. Then, the colon was cut within the range from the middle of the transverse colon to the hepatic flexure, including the poor color area, using Signia. Functional end-to-end anastomosis was conducted for reconstruction. In stomach reconstruction, Billroth II reconstruction was performed. The total surgical time was 464 min, and the estimated volume of blood loss was extremely low.

## Clinical outcomes

**Fig. 6:**
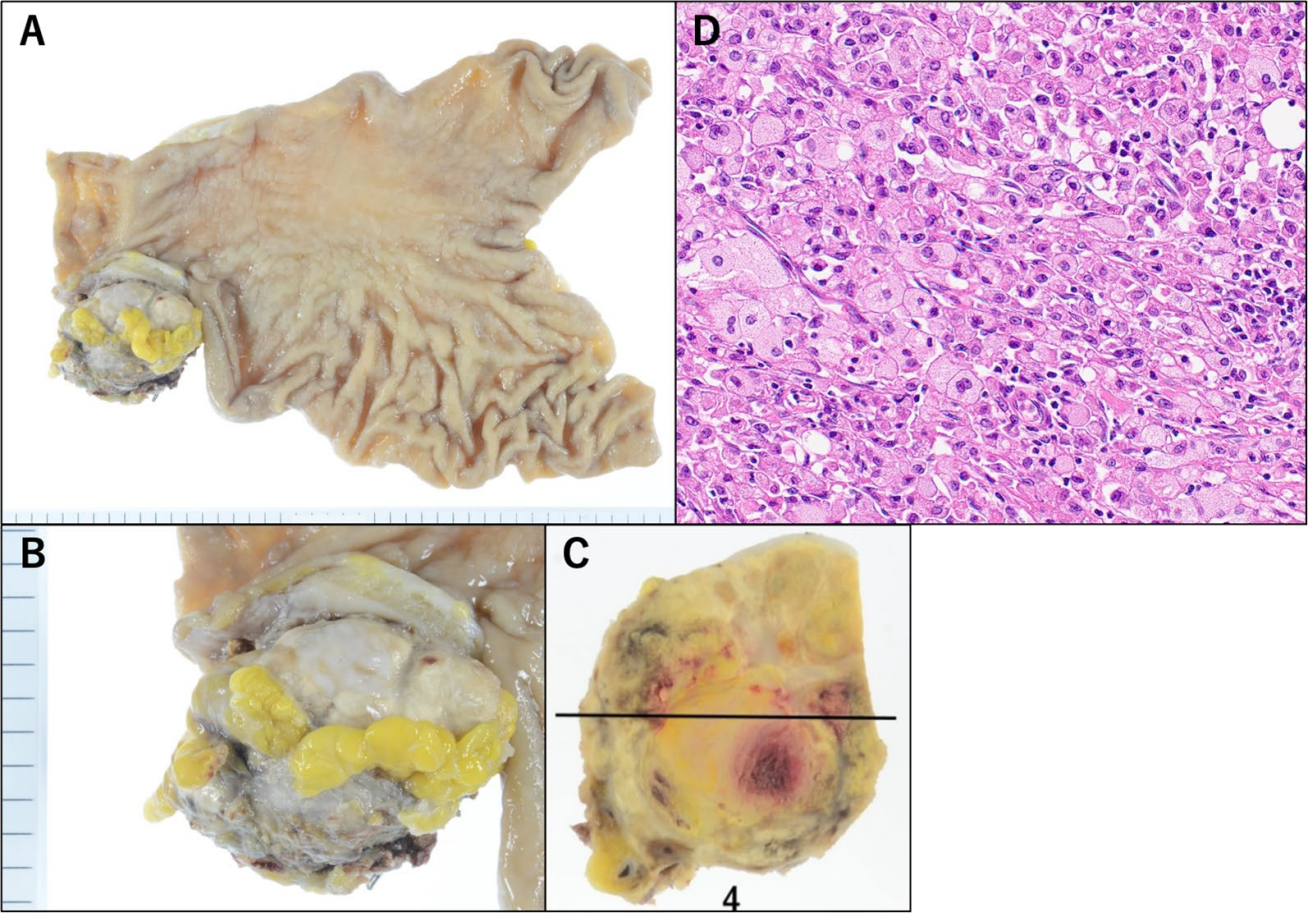
Regarding the surgical specimen (**A**), in terms of histology, the nodular lesion (**B**, **C**) was believed to be a lymph node. Gastric cancer had disappeared pathologically including the lymph node (**D**)

On postoperative day (POD) 3, CT scan showed an infiltrative shadow, which might be indicative of aspiration pneumonia in both lower lungs. Hence, antibiotic treatment was started. The patient was admitted for oral intake on POD 6. The patient was treated for aspiration pneumonia after changing antibiotic treatment from the intravenous to the oral route because of improvement. The complication was classified as grade 2 based on the Clavien–Dindo classification system. The patient was discharged from our hospital on POD 21 because of improvement in his general condition with a PS score of 1. Histopathological examination revealed that the huge mass in CECT had shrunk and was seen as a pyloric nodule, his gastric cancer had disappeared except for the scar with high-grade fibrosis in the submucosal layer (Fig. 6A). Moreover, there were no evident malignant findings. In terms of histology, the nodular lesion was believed to be a lymph node (Fig. 6B, C), which might have been invaded by cancer. However, viable tumor cells were not detected, and only necrosis and high-grade macrophage invasion were observed (Fig. 6D). Due to the absence of metastasis in other parts of the specimen, the condition was classified as grade 3 according to the histological treatment efficacy criteria. His quality of life improved significantly due to improved oral intake in addition to his general condition after discharge from the hospital. The patient was followed-up regularly with blood tests, CT scan, and EGD. However, no recurrence was observed at 2 years after surgery.

## Discussion

Gastrectomy is the standard surgical treatment for advanced-stage gastric cancer, and pancreatoduodenectomy is also considered for gastric cancer with pancreatic and duodenal invasion. However, pancreatoduodenectomy is rarely performed for gastric cancer because of significant morbidity and mortality and poor prognosis [7, 8]. Although recent reports have shown that the long-term prognosis is improving [9–12], the efficacy of pancreaticoduodenectomy for gastric cancer is limited, and the mortality rate of gastric cancer is still high. If the resection margin is positive, pancreaticoduodenectomy may be considered in younger patients without noncurative factors. However, in older patients, as in this case, the benefit is more limited, and the risk is higher [5, 6]. In this case, chemotherapy could prevent pancreaticoduodenectomy and facilitate gastrectomy, resulting in a less invasive treatment.

Laparoscopic surgery is becoming the standard treatment for advanced-stage cancer; however, it is still not common in T4b cases. Yu Pan et al. reported that there is no difference in the volume of blood loss and surgical time. The complication rate and length of hospital stay of elderly patients are longer than those of younger patients [13]. Hence, laparoscopic surgery has several advantages considering the invasiveness of the procedure. The minimally invasive approach for advanced-stage gastric cancer with multiple invasions to other organs can enable us to make a minor adjustment in the cutting line via the magnification effect and to resect surrounding organs within a minimal range.

In Japan, postoperative chemotherapy is the standard treatment for stage II/III gastric cancer, and several cases of postoperative chemotherapy have been collected [14, 15]. However, it challenging to sufficiently administer chemotherapy after surgery in older patients with gastric cancer because of decreased oral intake. Moreover, in some cases, postoperative chemotherapy is impossible because of complications. Although preoperative chemotherapy for locally advanced-stage gastric cancer is not common yet [16], it is easier to administer sufficient chemotherapy preoperatively compared with postoperative chemotherapy and can improve the cure rate [17].

The use of chemotherapy should be reduced or discontinued in some cases based on renal function and general condition. However, if the patient’s poor general condition is attributed to the tumor, chemotherapy may improve the general condition [18]. In this case, when the patient was initially referred to our department, chemotherapy and surgery were considered challenging. However, PS was believed to be declining because of cancer progression, and chemotherapy was administered in anticipation of surgery, which improved the patient’s PS and made surgery possible. It was a marginal lesion that may or may not be resectable, and there may have been an aspect of induction chemotherapy. In addition, the combination of rehabilitation and nutritional management using oral nutritional supplements in the hospital may have contributed to the improvement in his general condition.

In the present case, the patient developed aspiration pneumonia after surgery. Although the patient's symptoms improved with systemic management using antibiotics, the postoperative immunocompromised state of the patient by preoperative chemotherapy may put him at risk for various complications, including aspiration pneumonia. The risk of complications is expected to increase with advancing age, and complications may also reduce postoperative systemic status, leading to decreased ADL and a poorer long-term prognosis.

Considering that the patient was able to ingest orally, SOX was started as preoperative chemotherapy in this case. However, if oral intake is difficult, FOLFOX may be selected as preoperative chemotherapy. Laparoscopic distal gastrectomy with combined resection of the transverse colon was performed; however, if the patient had severe duodenal and pancreatic invasion and the general condition allows it, pancreaticoduodenectomy should be considered. However, if the duodenal or pancreatic invasion is so severe that gastrectomy is difficult, it may be necessary to focus on postoperative chemotherapy. There is no sufficient evidence on the outcomes, advantages, and disadvantages of preoperative chemotherapy. Hence, more cases should be evaluated in the future.

## Conclusion

After actively administering preoperative chemotherapy, radical surgery with maximum preservation of the surrounding organs using the minimally invasive approach can be performed for locally far advanced-stage gastric cancer in a patient with poor general condition. Chemotherapy can improve performance status, thereby enhancing curative effect and reducing resection extent. Therefore, preoperative chemotherapy can be useful even in patients with frailty who cannot tolerate extended surgery.

## Data Availability

None.

## Abbreviations

EGD: Esophagogastroduodenoscopy
CECT: Contrast-enhanced computed tomography
PET/CT: Positron emission tomography/computed tomography scan
PS: Performance status
SOX: S-1 and oxaliplatin
ASA-PS: American Society of Anesthesiology Physical Status
FDG: ^18^F-fluorodeoxyglucose
POD: Postoperative day
